# A flexible dual-band 4 × 4 MIMO antenna for 5G mm-wave 28/38 GHz wearable applications

**DOI:** 10.1038/s41598-024-65023-2

**Published:** 2024-06-21

**Authors:** Rakesh N. Tiwari, Deepti Sharma, Prabhakar Singh, Pradeep Kumar

**Affiliations:** 1 Department of Electronics and Communication Engineering, Madanapalle Institute of Technology and Science, Madanapalle, Andhra Pradesh 517325 India; 2 Department of Electronics and Communication Engineering, G.L. Bajaj Institute of Technology and Management, Greater Noida, Uttar Pradesh 201306 India; 3 Physics Division, School of Basic Sciences, Galgotias University, Greater Noida, Uttar Pradesh 203201 India; 4 Discipline of Electrical, Electronic and Computer Engineering, University of KwaZulu-Natal, Durban, 4041 South Africa

**Keywords:** Electrical and electronic engineering, Engineering

## Abstract

This paper presents a novel, dual-band, four-port multi-input–multi-output (MIMO) antenna for 28/38 GHz millimeter wave 5G wearable applications. In the proposed work, we have used a novel design approach to get the dual-band behavior from a MIMO design with a small footprint of 18 × 8.5 × 0.25 mm^3^. For this purpose, each MIMO element is designed as a composite form of a circular and elliptical structure connected with a narrow strip and fed by a tapered feedline. The peak realized gains and total efficiencies of the antenna, evaluated in free space, are 4.15 dBi, 7.73 dBi and 80.13%, 85.44% at 28 GHz and 38 GHz frequencies, respectively. To appraise the thorough behavior of the MIMO antenna, we have evaluated all the parameters of the antenna: Envelope Correlation Coefficient (ECC), Diversity Gain (DG), Mean Effective Gain (MEG), Channel Capacity Loss (CCL), and Total Active Reflection Coefficient (TARC), and found them satisfactory. Channel capacity of the antenna at SNR = 20 dB is found to be 21.61 bps/Hz. For wearable applications, the proposed 4-port MIMO antenna is designed on a flexible Rogers 3003 substrate, and the performance is checked by evaluating bending analysis. The safety of the antenna is verified by analyzing the 1 g/10 g SAR at 28/38 GHz and the corresponding average SAR values are 0.11/0.08 W/kg and 0.05/0.04 W/kg, respectively. All the average SAR values for the proposed MIMO antenna are within the acceptable limits according to FCC/ICNIRP standards.

## Introduction

The escalating field of wearable healthcare devices witness the people with numerous body-worn devices connected wirelessly might become common. The communication among wearable devices is likely to be highly proximal with a variable range of data rate requirements: from low data rate fitness bands to high data-rate augmented-reality headsets. MIMO antennas operating in the mm-wave range have significantly enhanced bandwidths and can support high data rates, spatial multiplexing, and spectral efficiency ^1–3^. Moreover, the low-latency and high data-rate communication capability of MIMO antennas make them useful in augmented reality and robotics applications ^4,5^.

A transparent 4-port MIMO design is reported with a footprint of 12 × 10 × 1.58 mm^3^ ^6^, and the antenna is operating in the frequency bands of 24.10–27.18 and 33–44.13 GHz. A dual-band MIMO antenna working at 28/38 GHz is presented in ^7^ using the concept of parasitically coupled patches. This design demonstrates good isolation of 34.5 and 27 dB at both the frequency bands, suitable for 5G MIMO systems. A dual-band 2 × 2 mm-wave MIMO antenna is designed using the rectangular patch tapered along the centre in a semi-circle configuration ^8^. Both the radiating elements are placed in an orthogonal position to minimize the coupling up to − 20 dB at both the operating bands of 28/38 GHz. A 4 × 4 MIMO antenna with dimension 30 × 30 × 1.575 mm^3^ is designed in ^9^ and operates in the frequency band of 26.16–29.72 GHz for 5G applications. Isolation of more than 29 dB was achieved by the two-iteration technique in the defected ground plane. Shorting pin-loaded patch antenna with an H-shaped slot is reported in ^10^ to operate independently at 28 and 38 GHz. This design exhibits antenna bandwidth of 3.8 and 9.0% across two resonance frequencies. Frequency reconfigurable mm-wave antenna is suitable for 5G handsets and designed by integrating diodes in the patch antenna ^11^. Varactor diodes are used to tune the operating frequency band from 23.2 to 30.2 GHz and the shorting pins improved the antenna efficiency. Pin-diodes can also be used in the MIMO antenna to alter the radiation state between linear polarization and circular polarization ^12^. A circularly polarized 4 port MIMO antenna is reported for on body applications with measured axial ratio bandwidth of 1190 MHz ^13^. This design utilized truncated patch and stub loaded defected ground plane with overall dimension of 40 × 40 × 1.6 mm^3^. In ^14^, a dual port dual band antenna is reported with radiating elements of hexagonal shape placed 180° with each other in MIMO design. This antenna covers the frequency bands of 25–29 GHz and 36–41 GHz with corresponding isolation > 25 dB. A similar approach is used in ^15^ to achieve an omnidirectional 2 × 2 MIMO antenna operating at 28 GHz. Another dual-band MIMO antenna reported in ^16^ with a CPW-fed slot-loaded configuration for mm-wave application in the frequency bands 27.5–30.9 GHz and 37.3–44.6 GHz. A high gain characteristic of the mm-wave antenna is achieved by a two-pronged fork-shaped antenna (in 8 × 8, 8 × 16, and 8 × 32 configurations) ^17^. These array antennas operate between 37.3 to 39 GHz for 5G mm-wave base stations. The isolation improvement in mm-wave MIMO antenna elements can be achieved by adopting a specific shape of the patch, self-isolation technique, and the H-plane decoupling method ^18–20^. Further, the cross-shaped MIMO design with a split ring resonator is reported, and the improved isolation values were 35 and 33 dB at frequencies of 24.5 and 28.5 GHz, respectively ^21^. A decoupling structure consisting of a zig-zag pattern improves the isolation in 4 × 4 MIMO elements ^22^ up to 40 dB across the operating band 27.5–28.5 GHz. An isolation improvement by ground plane with a square slot in the 4-port MIMO design is reported in ^23^. The antenna consists of composite radiating elements with an inset-fed microstrip line to achieve dual-band response at 28/38 GHz. In ^24^ EBG structure is used to reduce the mutual coupling upto − 40 dB at 24 GHz. With the EBG structure, this quad port mm-Wave MIMO design has achieved the peak gain of 6.0dBi and found suitable for wearable applications.

In Table 1, a comparison of previously reported mm-wave antennas is presented. MIMO antennas proposed in ref. ^9,17,18,21–23^ are 4-port antennas, but all these antennas have large footprints as compared to the 2-port antennas ^3,7,8,15^. The 2-port MIMO antenna proposed in ^8^ has the lowest gain (1.83 dBi), and the 4-port antenna of Ref.^22^ has the highest gain (12 dBi) as compared to the other antennas, but the size of this antenna is quite large. In addition to that, it should be noted that most of the antennas have reported only ECC and MEGs while other important MIMO parameters such as CCL, Channel Capacity (CC), Cumulative Distribution Function (CDF), bending analysis, SAR analysis, and link budget were not evaluated.

**Table 1:** Performance comparison with other MIMO antennas.

Ref./year	No. of ports	Volumetric size (mm^3^)	Freq. bands (GHz)	Isolation (dB)	Peak gain (dBi)	ECC	MEG (dB)	CCL (bits/s/Hz)	Bending analysis	SAR (1/10 g)	Link budget
^3^ (2022)	2	11.4 × 5.3 × 0.8 (48.34)	29 (center)	> 36	6	< 0.001	NR	NR	NR	NR	NR
^7^ (2022)	2	7.5 × 8.8 × 0.25 (16.50)	28/38	> 20	6.6	value not given	NR	NR	NR	NR	NR
^8^ (2019)	2	26 × 14 × 0.38 (138.32)	26.65–29.2/36.95–39.05	> 20	1.83	0.001	NR	< 0.4	NR	NR	NR
^9^ (2022)	4	30 × 30 × 1.575 (1417.5)	26.4–29.75	> 30	7.1	0.0005	− 6	0.15	NR	NR	NR
^10^ (2020)	1	25 × 15 × 0.762 (285.75)	27.7–28.7/36.8–40.2	NR	9.0	NR	NR	NR	NR	NR	NR
^14^ (2021)	2	26 × 11 × (value not given)	25–29/37–41	> 25	5.7	10^–4^	NR	NR	NR	NR	NR
^16^ (2022)	2	40.4 × 20 × 1.136 (917.89)	27.6–30.3/33.4–40.4	> 20	8	0.0001	1 (ratio)	< 0.1	NR	NR	NR
^17^ (2021)	4	47.4 × 32.5 × 0.51 (785.66)	36.83–40.0	> 25	6.5	0.001	NR	< 0.6	NR	NR	NR
^18^ (2022)	4	24 × 24 × 0.254 (146.30)	22.43–31.66	> 25	5.66	0.008	− 2.17	NR	NR	NR	NR
^21^ (2023)	4	40 × 72 × 0.58 (1670.40)	23.7–25.55/27.8–31.8	> 33	7.9	< 10^–4^	NR	NR	NR	NR	NR
^22^ (2022)	4	30 × 35 × 0.787 (826.35)	27.5–28.5	> 40	12	< 0.0003	NR	< 0.4	NR	NR	NR
^23^ (2023)	4	26 × 26 × 0.25 (169.00)	27.7–28.3/ 37.7–38.3	> 33	8.1	< 0.0005	NR	< 0.0064	NR	NR	NR
Prop. work	4	18 × 8.5 × 0.25 (38.25)	27.76–28.48/37.69–38.19	> 20	7.73	< 0.03	− 6	< 0.15	Yes	Yes	Yes

*NR* Not reported.

Therefore, in order to overcome the above issues, in this paper, we have proposed an ultra-compact 4-port MIMO antenna with good gain, and we have evaluated all the parameters of the MIMO antenna such as ECC, DG, MEG, CCL, TARC, CC and CDF. Also, in order to prove the effectiveness of the proposed 4-port MIMO antenna for wearable applications, we have performed detailed bending analysis of the antenna. Subsequently, safety of the wearer is also taken into account by performing 1 g/10 g SAR analysis, which is within acceptable limit according to the FCC/ICNIRP standards. Lastly, link budget is also evaluated at low transmitted power of 10 mW (10 dBm), which shows that the proposed MIMO antenna can communicate up to 60 m of distance.

### Antenna design

**Figure 1:**
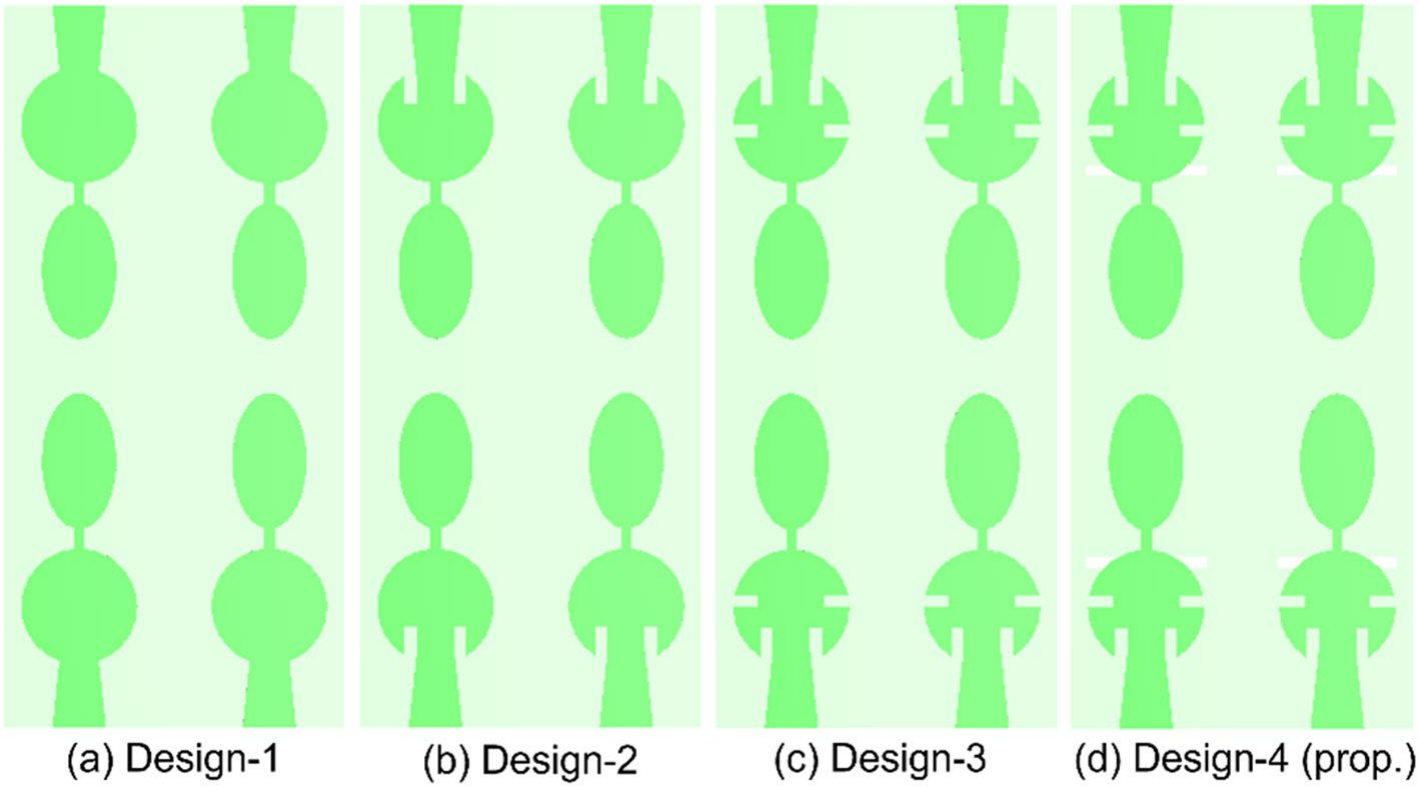
Design evolution of 4 × 4 MIMO antenna.

**Figure 2:**
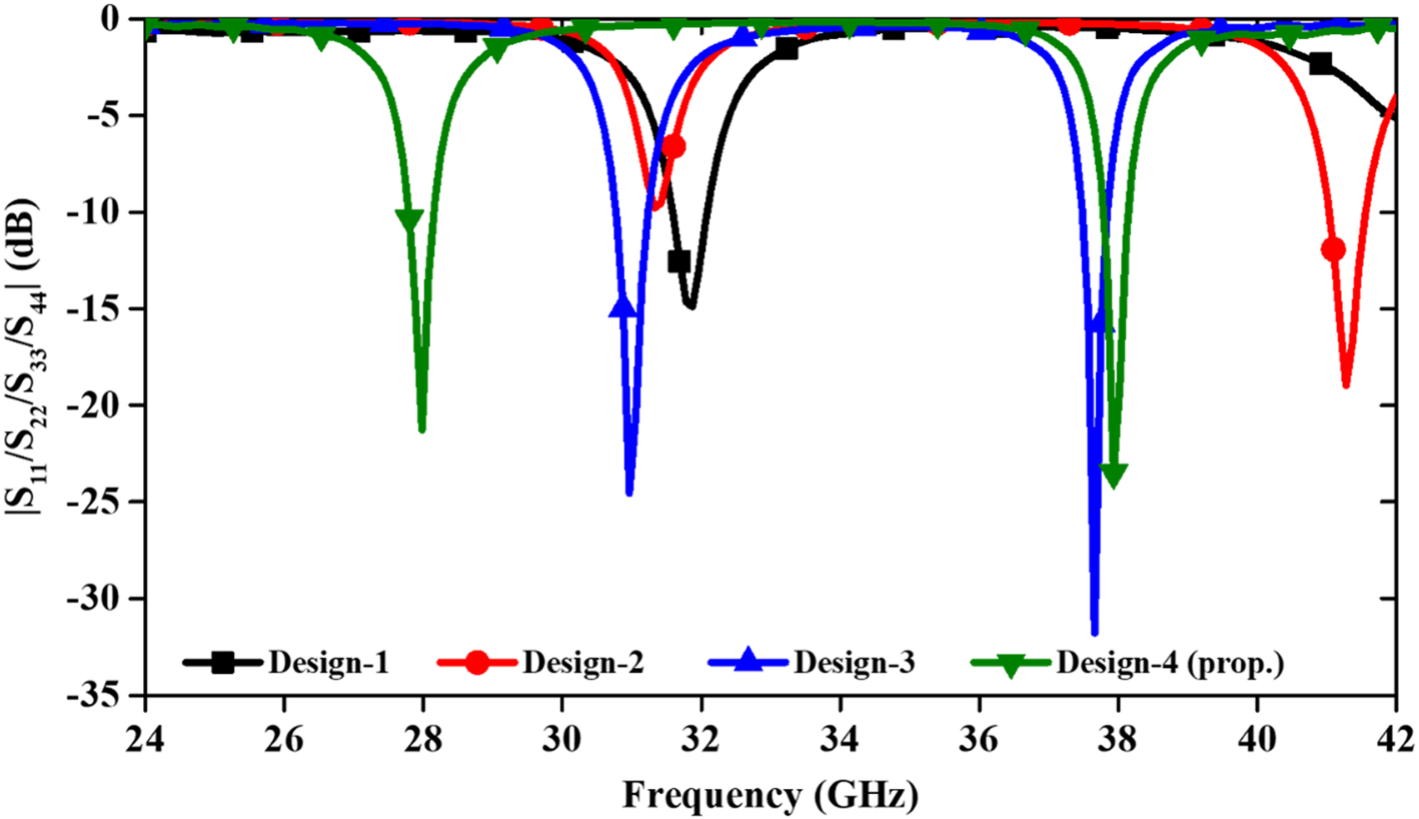
Comparison of the reflection coefficients for different MIMO antennas.

The structure of each MIMO antenna element is selected such that the antenna can resonate in two different frequencies. In this process, initially, a composite structure using a circular patch connected with an elliptical shaped patch is designed. The coupled resonator is excited with a tapered feedline (Design-1 in Fig. 1). However, the higher resonance frequency is not obtained as per the expectation. Therefore, in Design-2, the microstrip feed line is modified as inset fed and the higher resonance frequency shifted towards lower side (41.2 GHz), due to the increased current path from feed to the curvature of patch. Further, in third step (Design-3), a pair of slots is etched in the circular patch, which further shifted the higher resonance at 38 GHz, yet the lower resonance is not in the desired frequency range. Thus, to obtain both the frequencies at required range, a narrow rectangular slot is etched in the ground plane just below the top of the circular patch (design-4). This final design optimized the resonances of the MIMO antenna to resonate at 28/38 GHz. The S-parameter results for all antenna designs can be seen in Fig. 2. The overall dimension of the MIMO antenna is 18 × 8.5 × 0.25 mm^3^. The flexible substrate (Rogers RO 3003) with dielectric constant 3.0 and loss tangent 0.001 is used for wearable electronics.

**Figure 3:**
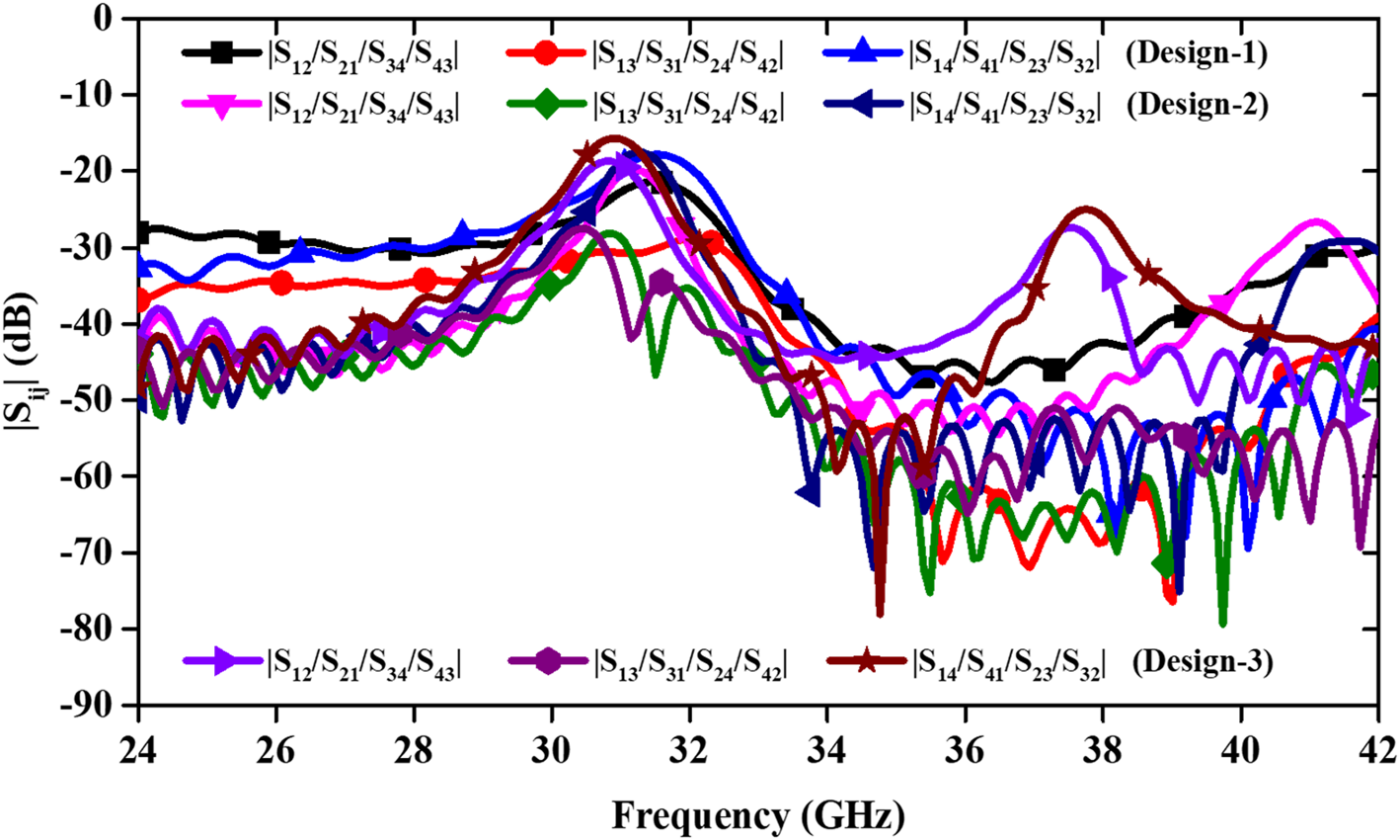
Comparison of the transmission coefficients for different MIMO designs.

**Figure 4:**
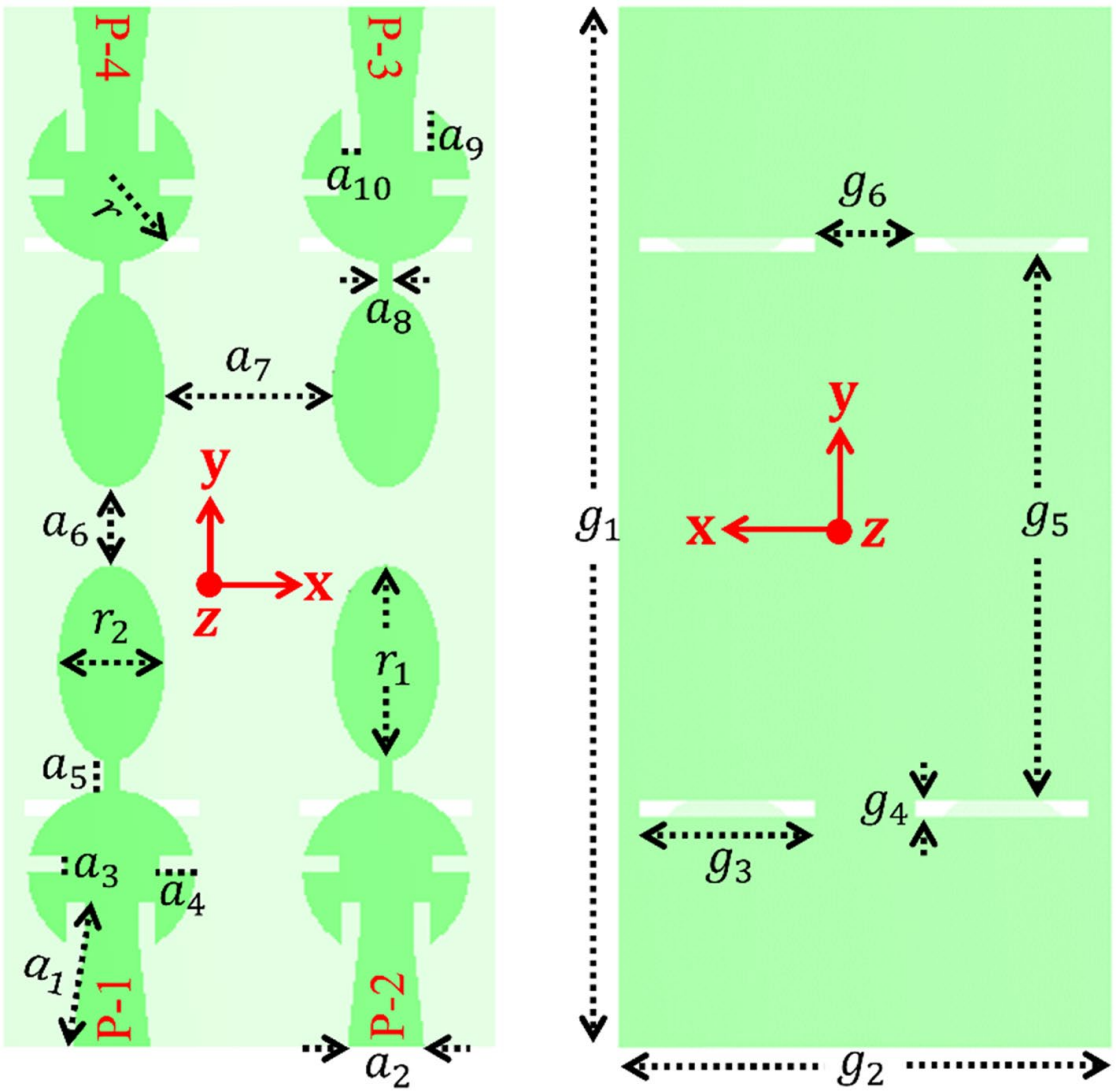
Optimized 4 × 4 MIMO antenna design parameters.

The isolation between the antenna ports is close to 20 dB in all the design steps of evolution, as shown in Fig. 3. While designing the 4-port MIMO antenna, it was observed that λ_0_/4 and λ_0_/8 spacing between the horizontal and vertical antenna elements, respectively, provides isolation of 20 dB, and if this spacing reduces, isolation deteriorates. The proposed MIMO antenna design is illustrated in Fig. 4. Circular and elliptical sections are connected together with a narrow strip, as shown in Fig. 4. This composite structure of each antenna element in the MIMO design resonates in dual frequency bands at 28/38 GHz. The slot created in the ground plane adds the capacitance and affects the lower resonance (28 GHz). The design specifications of the proposed antenna are given in Table 2.

**Table 2:** Design specification of the proposed MIMO antenna (unit: in mm).

Parameter	${a}_{1}$	${a}_{2}$	${a}_{3}$	${a}_{4}$	${a}_{5}$	${a}_{6}$	${a}_{7}$
Value	2.51	1.33	0.30	0.63	0.50	1.34	2.95
Parameter	${a}_{8}$	${a}_{9}$	${a}_{10}$	$r$	${r}_{1}$	${r}_{2}$	${g}_{1}$
Value	0.26	0.73	0.30	1.45	1.70	0.93	18.00
Parameter	${g}_{2}$	${g}_{3}$	${g}_{4}$	${g}_{5}$	${g}_{6}$	–	–
Value	8.50	3.00	0.25	9.50	1.75	–	–

**Figure 5:**
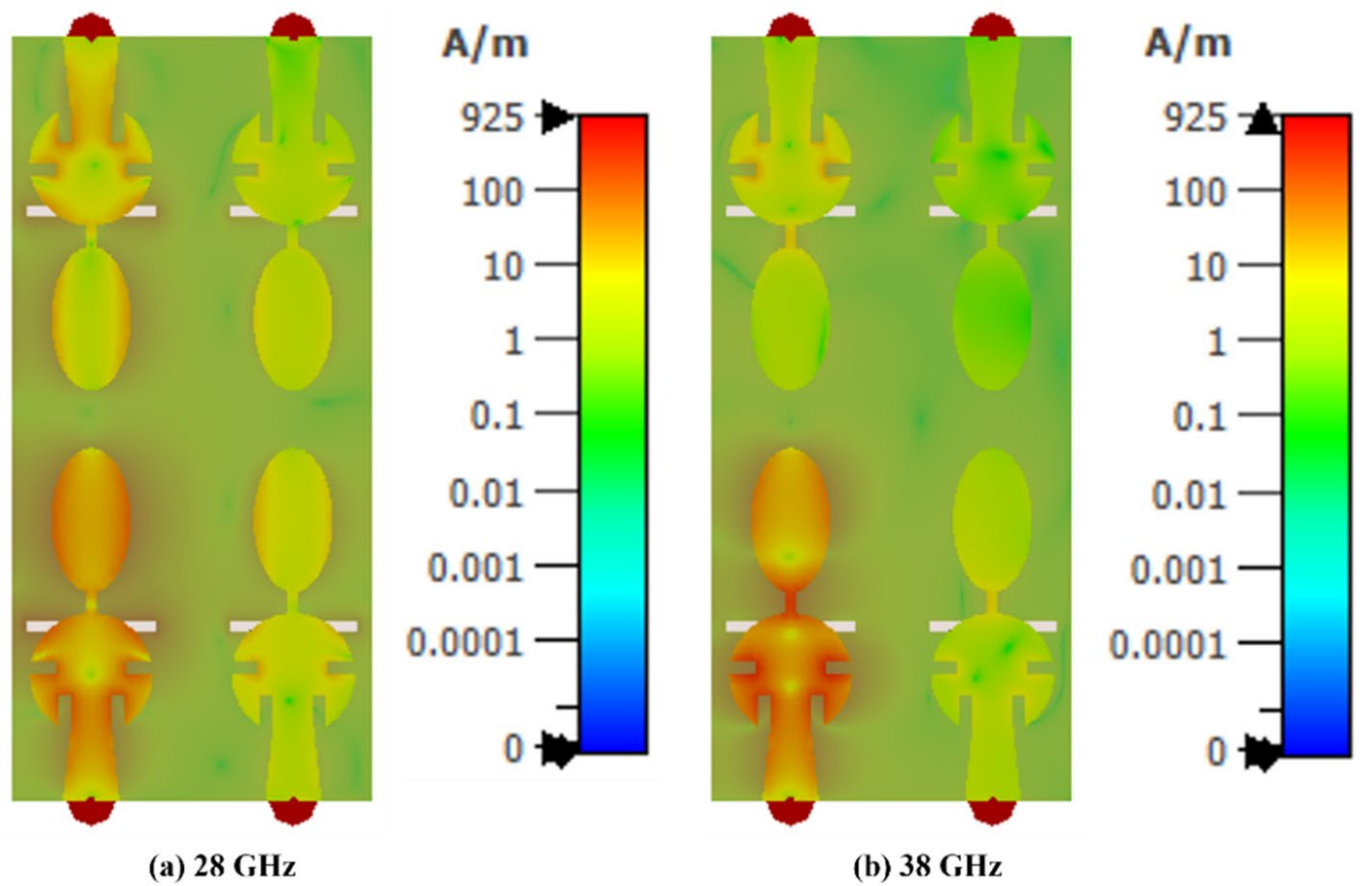
Current distribution on the MIMO antenna at 28 and 38 GHz.

**Figure 6:**
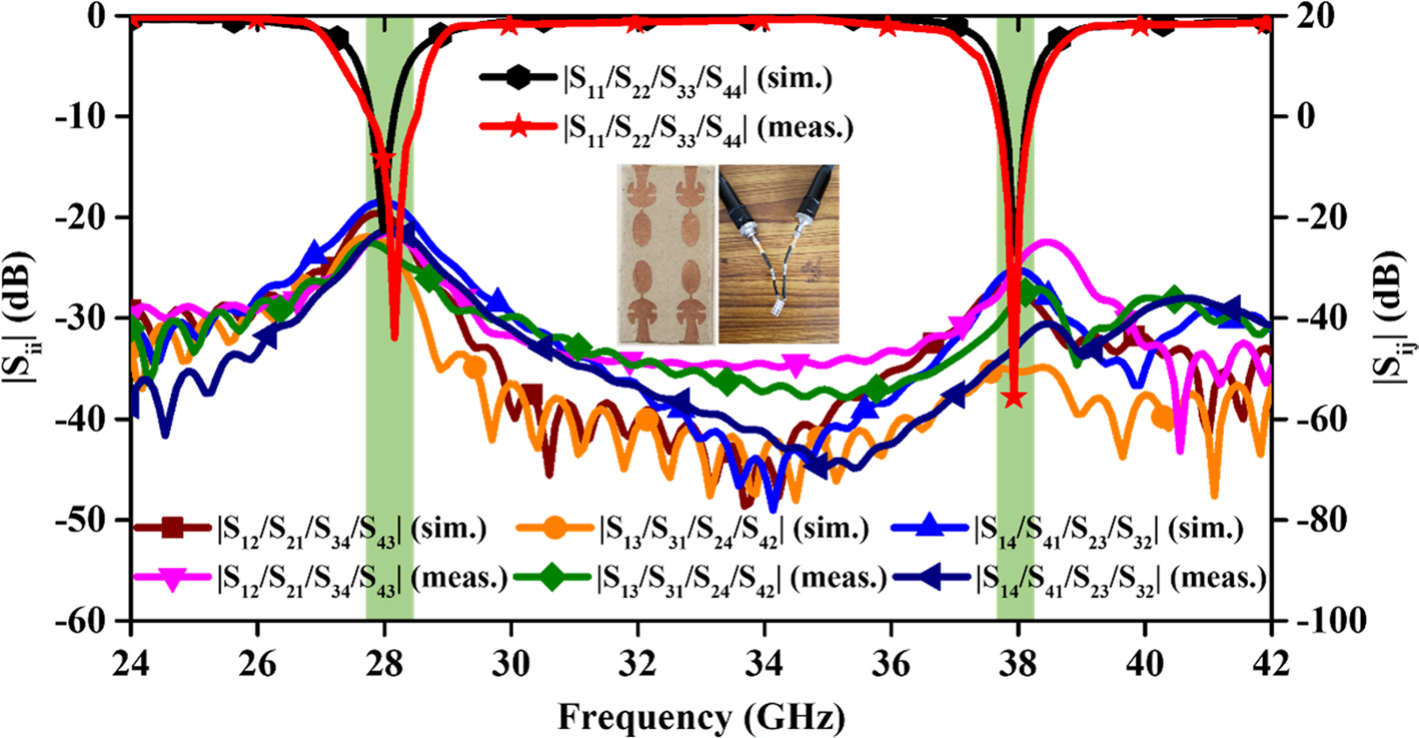
Comparison of simulated and measured S-parameter results.

The current distribution of the designed MIMO antenna (at 28 GHz and 38 GHz) is shown in Fig. 5. It can be observed that the current density is high on the elliptical patch and on the slot in the ground plane, affecting the lower resonance at 28 GHz. The pair of slots etched in the circular patch marginally affects the 38 GHz resonance, as shown in Figs. 2 and 5b. Furthermore, at 28 GHz, more coupling is observed as compared to higher frequency of 38 GHz and it can be seen from Fig. 6 (S_14_/S_41_/S_23_/S_32_). It may be explained based on the fact that MIMO antenna resonates at fundamental mode at 28 GHz and hence the surface current on the patch is higher. Consequently, the coupling with nearby radiating element is higher as compared to the case when the antenna is resonating at 38 GHz.

## Analysis of results

### Comparison of simulated and measured S-parameters

The fabricated prototype of the MIMO antenna alongwith the comparison of the simulated and measured S-parameters are shown in Fig. 6. The simulated |Sii| values for all the antenna ports are almost identical. The measured value of the scattering parameters has shifted a little higher side (might be due to fabrication tolerances), but still, it is covering the entire frequency band, efficiently. All the simulated values of transmission coefficients are in agreement with the measured values, as shown in Fig. 6.

### Peak gain and the total efficiency

**Figure 7:**
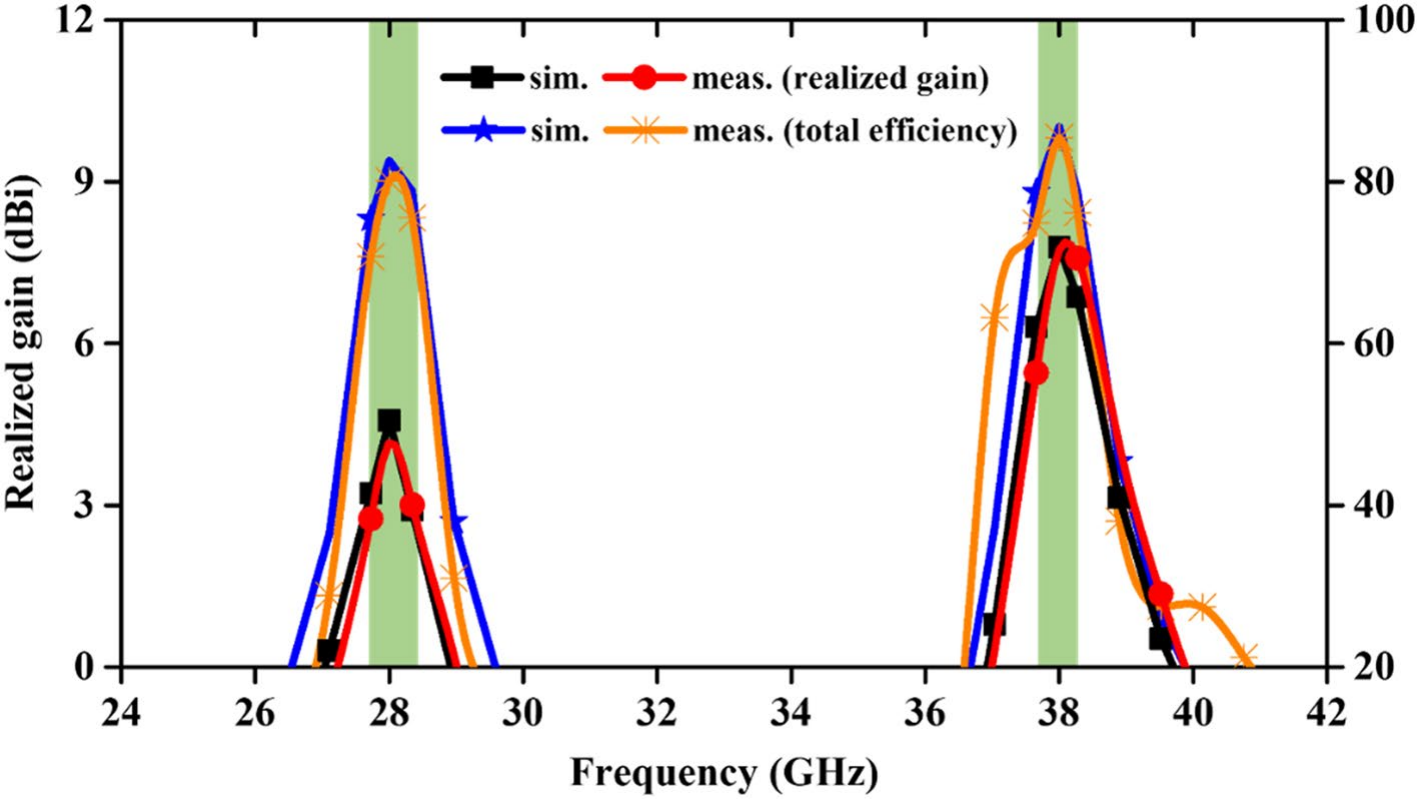
Realized gain and total efficiency of the antenna.

Further, the realized gain and total efficiency of the antenna across two operating bands are evaluated in free space and compared with the measured results (Fig. 7). From the graph, it can be found that the measured realized gain and total efficiency at 28 GHz are 4.15 dBi and 80.13% (simulated: 4.57 dBi and 82.56%) while at 38 GHz, it is 7.73 dBi and 85.44% (simulated: 7.79 dBi and 86.76%), respectively.

**Figure. 8:**
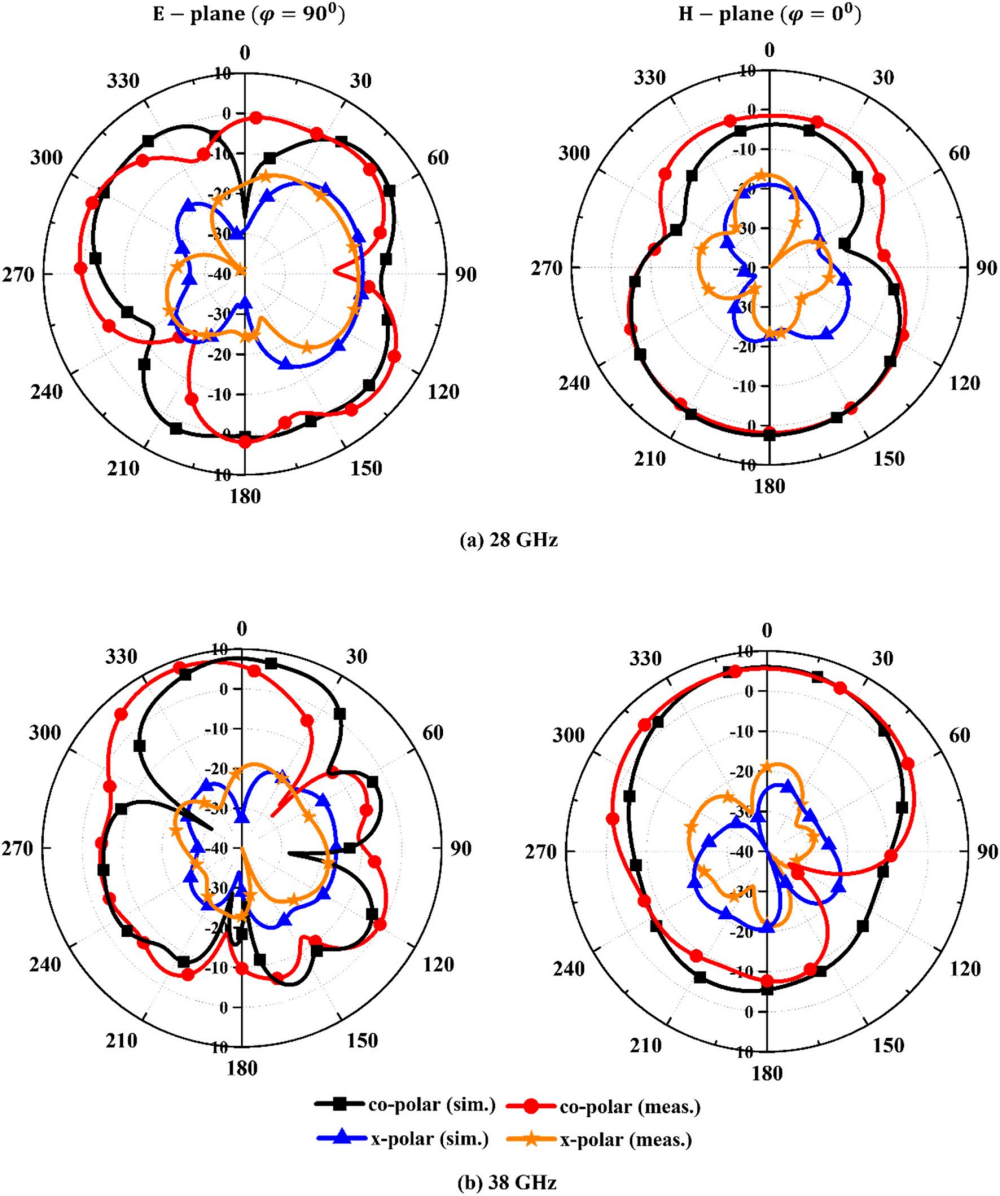
Radiation patterns of the MIMO antenna.

The far field radiation patterns at 28 and 38 GHz for elevation (phi = 90 degree) and azimuthal planes (phi = 0 degree) are depicted in Fig. 8. The co- and cross-polarization patterns are satisfactory at both the frequencies to be used in MIMO technologies.

### Analysis of diversity parameters of 4-port MIMO antenna

Along with the port isolation, the correlation analysis in MIMO antennas is also important to evaluate the MIMO antenna performance. Therefore, different diversity parameters such as ECC, DG, MEG, CCL, TARC, CC and CDF are presented in this section.

(i) ECC and DG: The maximum allowable value of ECC, as per the standards, is 0.5 and all the values more than 0.5 are not acceptable. The higher value of ECC increases the coupling between the MIMO antenna elements and hence causes the loss of transmitted power.

**Figure 9:**
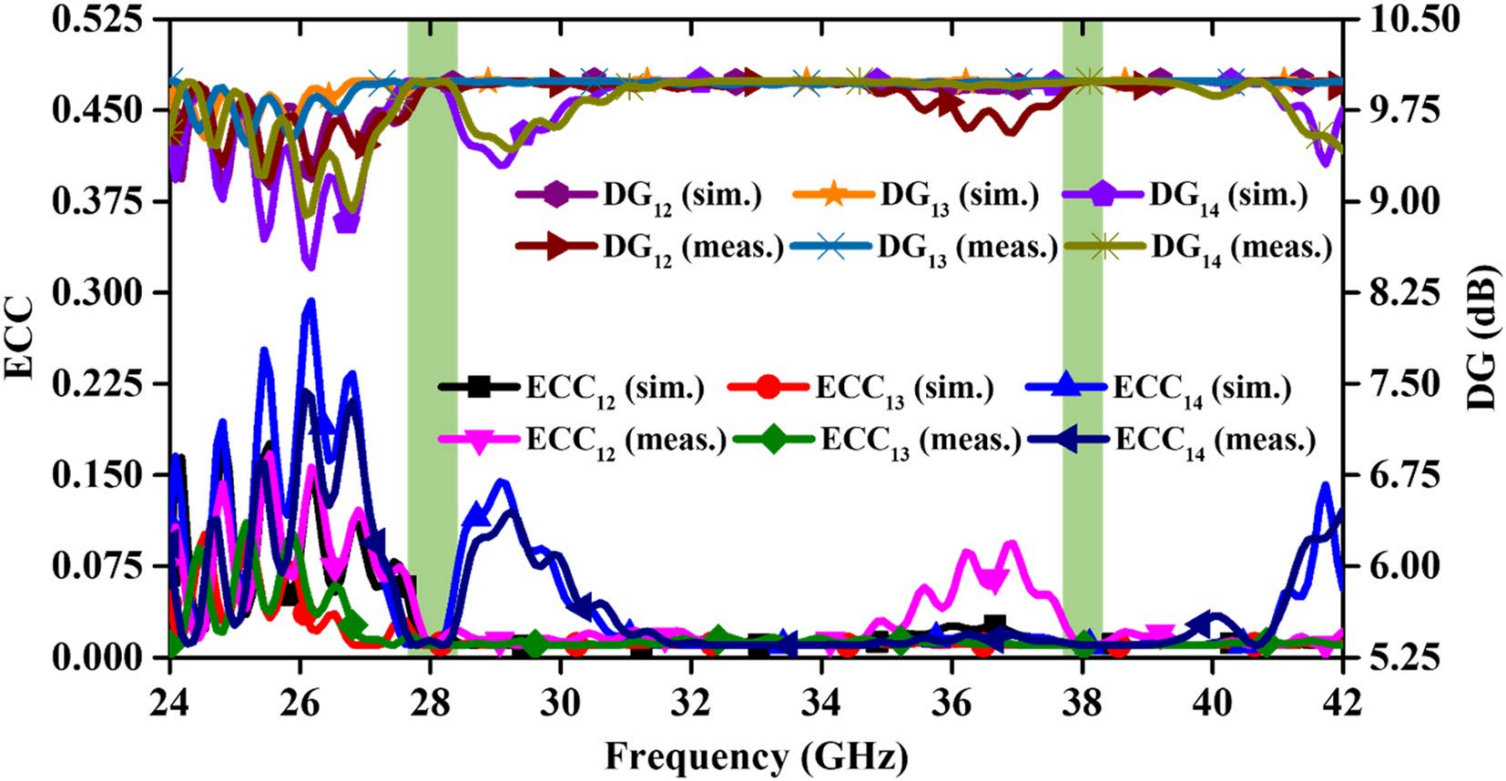
Simulated and measured ECC and DG of the MIMO antenna.

**Figure 10:**
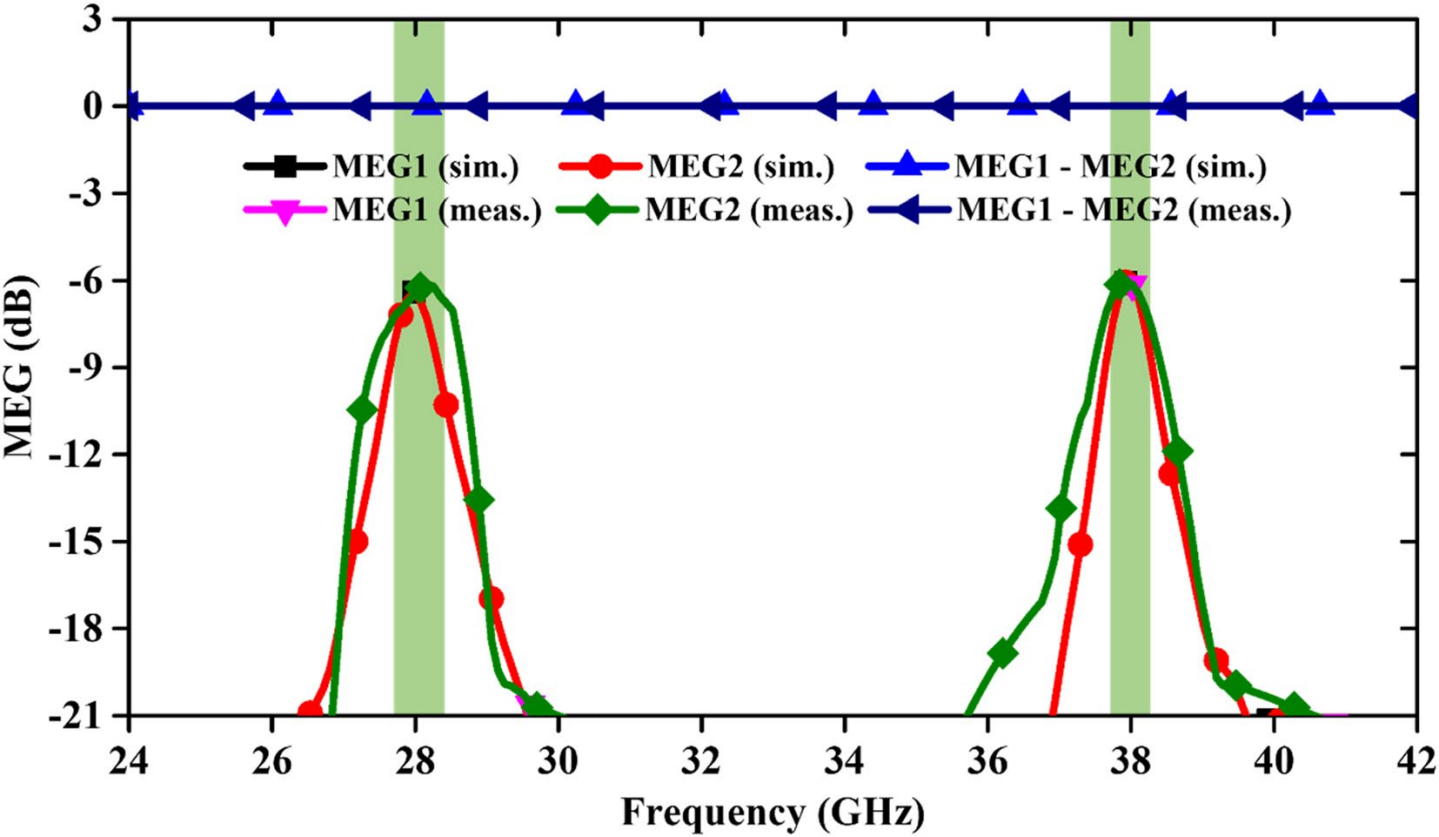
Simulated and measured MEG of the antenna.

**Figure 11:**
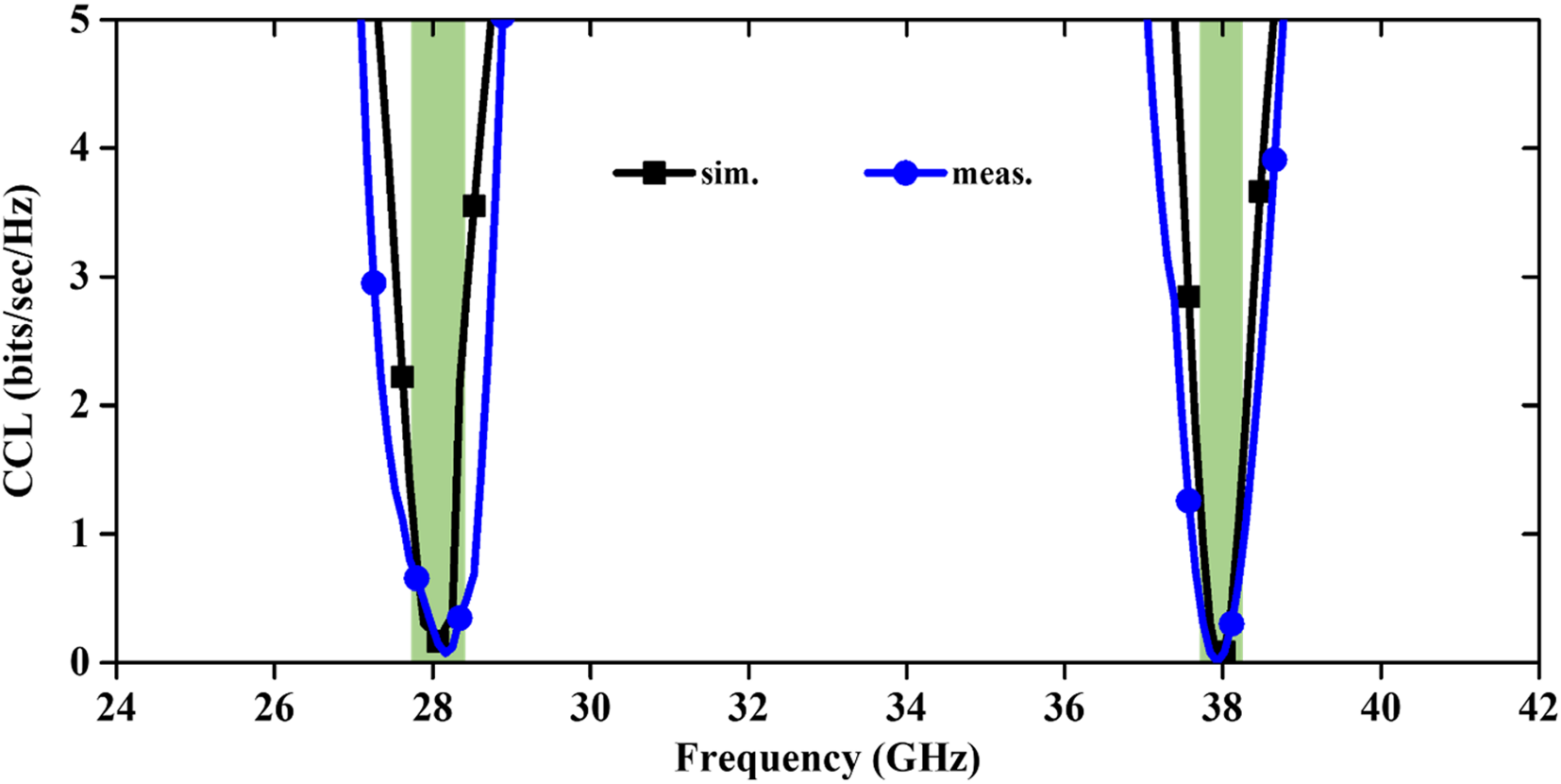
Simulated and measured values of CCL.

**Figure 12:**
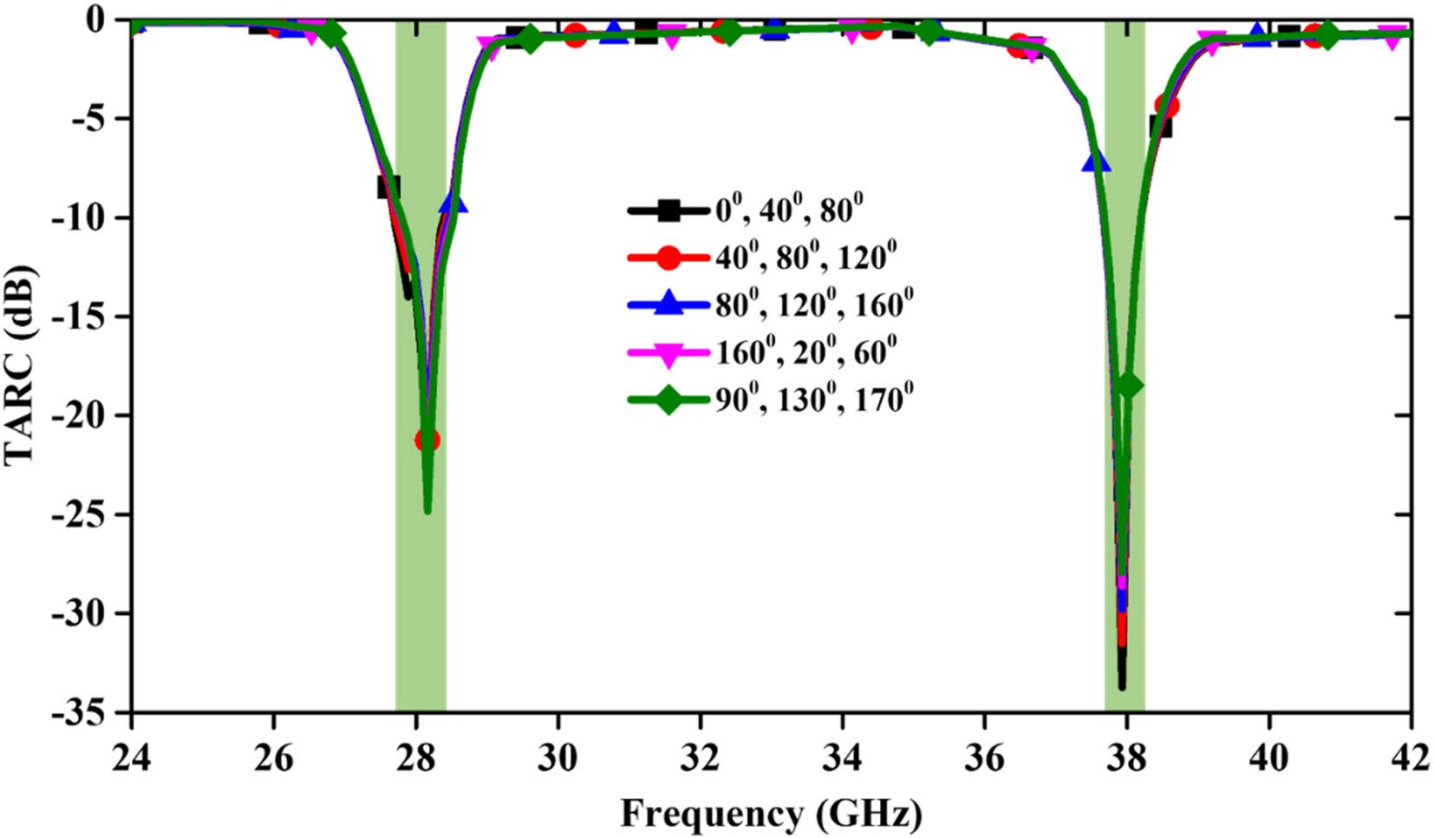
Calculated TARC at various phase angles.

The ECC values between different ports in the MIMO system can be calculated using ^25^.

1 $${ECC}_{\mathcal{r}\mathcal{s}}=\frac{{\left|\underset{0}{\overset{2\pi }{\int }}\underset{0}{\overset{\pi }{\int }}\left(XPR\cdot {E}_{\vartheta \mathcal{r}}{E}_{\vartheta \mathcal{s}}^{*}{P}_{\vartheta }+{E}_{\varphi \mathcal{r}}{E}_{\varphi \mathcal{s}}^{*}{P}_{\varphi }\right)\mathcal{d}\Omega \right|}^{2}}{\underset{0}{\overset{2\pi }{\int }}\underset{0}{\overset{\pi }{\int }}\left(XPR\cdot {E}_{\vartheta \mathcal{r}}{E}_{\vartheta \mathcal{r}}^{*}{P}_{\vartheta }+{E}_{\varphi \mathcal{r}}{E}_{\varphi \mathcal{r}}^{*}{P}_{\varphi }\right)\mathcal{d}\Omega \times \underset{0}{\overset{2\pi }{\int }}\underset{0}{\overset{\pi }{\int }}\left(XPR\cdot {E}_{\vartheta \mathcal{s}}{E}_{\vartheta \mathcal{s}}^{*}{P}_{\vartheta }+{E}_{\varphi \mathcal{s}}{E}_{\varphi \mathcal{s}}^{*}{P}_{\varphi }\right)\mathcal{d}\Omega }$$

where, $XPR={P}_{\mathcal{v}}/{P}_{\mathcal{h}}=1$, and XPR = cross polarization rate. In the proposed 4-port MIMO antenna, ECC values at two operating bands are calculated and illustrated in Fig. 9. It is to be noted that far-field patterns are used to calculate the ECC values and found to be 0.03 and 0.01 at 28 and 38 GHz, respectively. The ideal value of DG for completely isolated radiating elements is always 10 dB. In the proposed antenna, the DG values at two operating frequencies are quite close to the ideal value (9.87 dB) for different ports.

(ii) MEG: The MEG value of the mm-Wave MIMO antenna is calculated using far-field patterns and it can be expressed as ^26^
  2 $$MEG=\frac{{P}_{\mathcal{r}}}{{P}_{\mathcal{i}\mathcal{n}}}=\underset{0}{\overset{2\pi }{\int }}\underset{0}{\overset{\pi }{\int }}\left\{\frac{XPR}{1+XPR}\times {F}_{\vartheta }\left(\vartheta ,\varphi \right){P}_{\vartheta }\left(\vartheta ,\varphi \right)+\frac{1}{1+XPR}\times {F}_{\varphi }(\vartheta ,\varphi ){P}_{\varphi }(\vartheta ,\varphi )\right\}\text{sin}\vartheta d\vartheta d\varphi$$
  where, ${F}_{\vartheta }$ and ${F}_{\varphi }$ denote the power gain patterns of the MIMO antenna. MEGs and their differences across the dual operating bands are presented in Fig. 10, and at both resonance frequencies, it is almost − 6 dB.
(iii) CCL: Furthermore, the CCL of the antenna is evaluated and shown in Fig. 11. The CCL values at 28/38 GHz are again almost same i.e. 0.15 bits/s/Hz. The obtained CCL values are less than the defined practical value of 0.4 bits/s/Hz ^27^. All the calculated values are in good agreement with the measured values.
(iv) TARC: The TARC calculation of the MIMO antenna at different phase angles (0° to 170° with equal interval of 40°) of input signal is illustrated in Fig. 12. TARC is almost stable at two operating frequency bands, ensuring a stable MIMO design for 5G systems.
(v) Channel Capacity (CC): The MIMO antenna is preferred over the Single Input Single Output (SISO) antenna due to the increased CC without enhancing the frequency range and additional power to the system. More number of highly isolated antenna elements in the MIMO design is required to increase the channel capacity. The CC for N × N MIMO antenna (N is number of radiating elements) is expressed as ^28^
  3 $$CC={log}_{2}\left[det\left\{{I}_{{N}_{{R}_{x}}}+\frac{{\left(SNR\right)}_{avg.}}{{N}_{{T}_{x}}}H{H}^{*}\right\}\right]$$
  here, ${N}_{{T}_{x}}$ and ${N}_{{R}_{x}}$ are the numbers of transmitted and received antenna elements, respectively, ${I}_{{N}_{{R}_{x}}}$ is the identity matrix (${N}_{{R}_{x}}\times {N}_{{R}_{x}}$), $H=\left[{h}_{ij}\right]\in {C}^{{N}_{{R}_{x}}\times {N}_{{R}_{x}}}$ is the normalized channel matrix, and ${H}^{*}$ indicates the conjugate transpose matrix of H.

The “Kronecker” model ^29^ is used for calculating the channel matrix $H$ and it is expressed as:

4 $$H=\left(\sqrt{{\psi }_{R}} \right)G\left(\sqrt{{\psi }_{T}} \right)$$

where, ${\psi }_{T}$ and ${\psi }_{R}$ represent the transmit and receive correlation matrix, $G$ is the random matrix with Gaussian entries. The Eq. (4) can be used to calculate $\left(\fancyscript{u},\fancyscript{v}\right)$ entry of ${\psi }_{{R}_{x}}$.

5 $${\psi }_{{R}_{x}}^{\left(\fancyscript{u}, \fancyscript{v}\right)}=\frac{{\zeta }_{\fancyscript{u}\fancyscript{v}}}{\sqrt{{\zeta }_{\fancyscript{u}\fancyscript{u}}{\zeta }_{\fancyscript{v}\fancyscript{v}}}}$$

The value of ${\zeta }_{\fancyscript{u}\fancyscript{v}}$ can be calculated as ^30^:

6 $${\zeta }_{\fancyscript{u}\fancyscript{v}}=\underset{0}{\overset{2\pi }{\int }}\left[\Gamma {A}_{\fancyscript{u}\theta }\left(\frac{\pi }{2},\varphi \right){A}_{\fancyscript{v}\theta }^{*}\left(\frac{\pi }{2},\varphi \right)+{A}_{\fancyscript{u}\varphi }\left(\frac{\pi }{2},\varphi \right){A}_{\fancyscript{v}\varphi }^{*}\left(\frac{\pi }{2},\varphi \right)\right]d\varphi$$

here, ${A}_{\theta }$ and ${A}_{\varphi }$ = radiation field patterns of the $\fancyscript{u}$ and $\fancyscript{v}$ antenna elements, respectively. $\Gamma$ = cross-polarization discrimination of the receiving EM wave. Here, the value of $\Gamma$ is taken as 0 dB for the proposed MIMO antenna ^31^.

**Figure 13:**
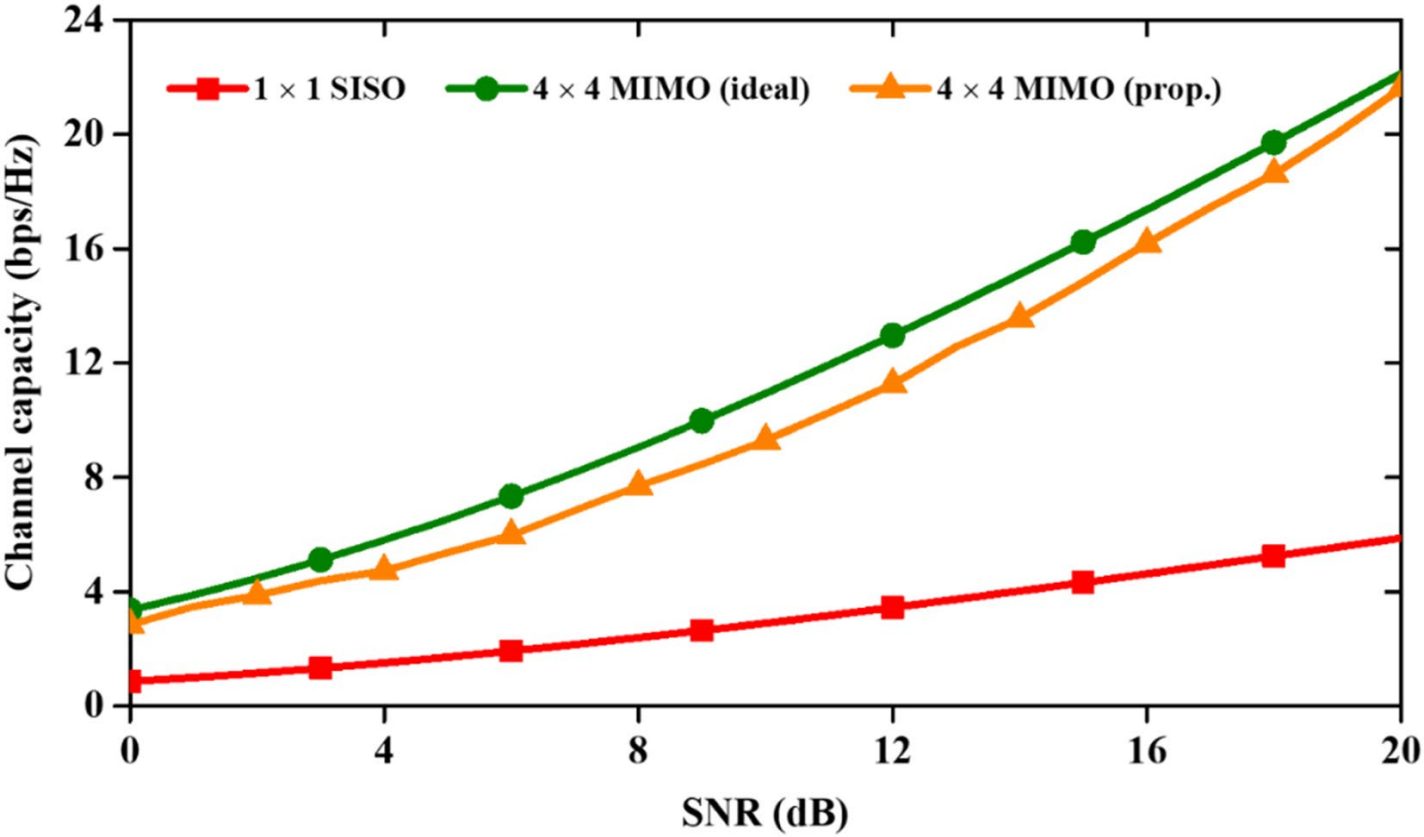
CC with respect to SNR of the proposed mm-wave MIMO antenna.

The variation of CC with signal-to-noise-ratio (SNR) is plotted in Fig. 13. The proposed 4-port MIMO antenna has a CC value (at SNR = 20 dB) of 21.61 bps/Hz, and an ideal SISO antenna has a channel capacity of 5.88 bps/Hz. Thus, the proposed antenna has enhanced the CC more than four times as compared to the SISO antenna. In addition to that, the CDF is calculated using the radiation patterns at 28 GHz.

**Figure 14:**
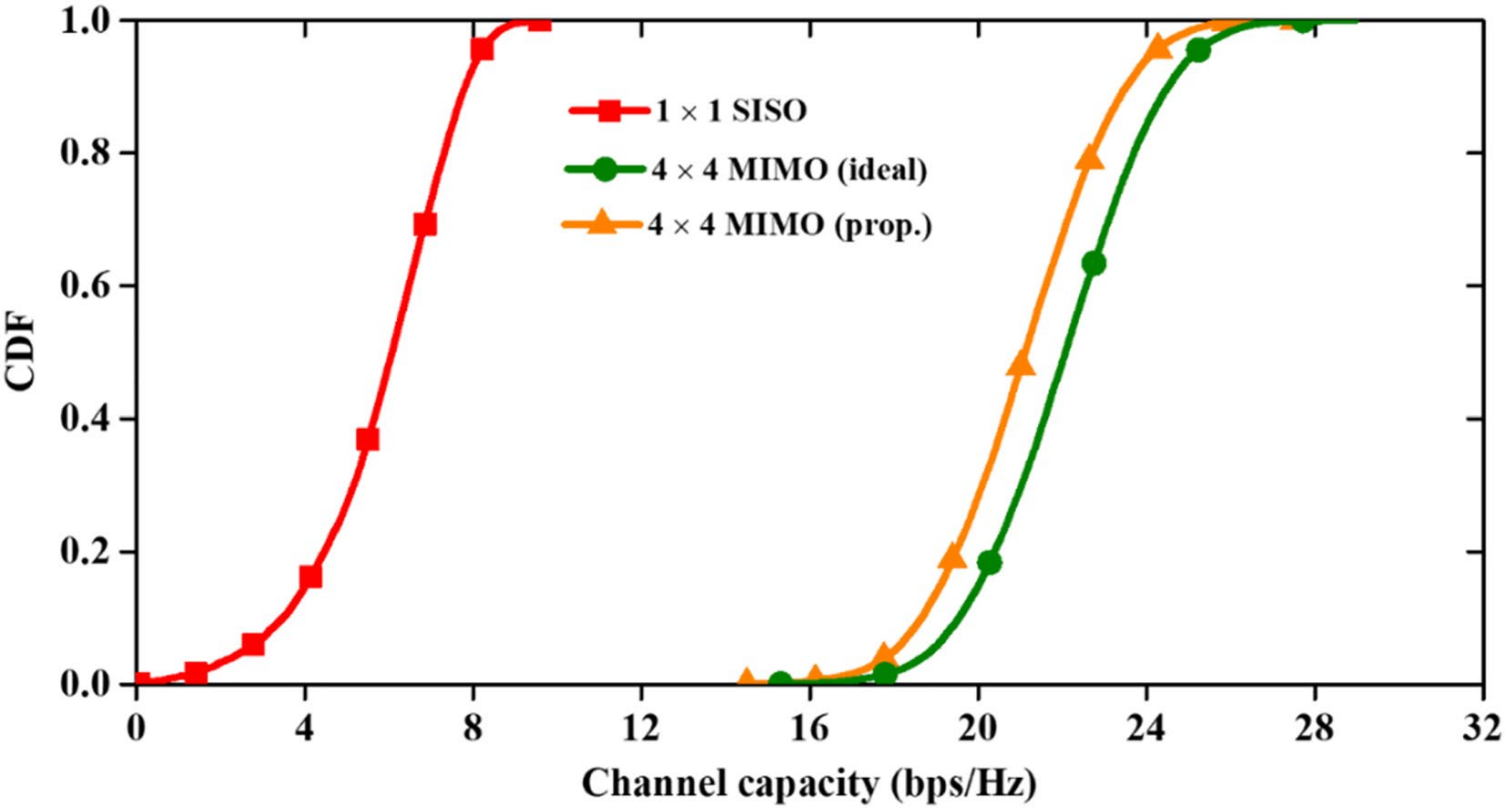
CDFs values of channel capacity in SISO, 4 × 4 MIMO (ideal) and 4 × 4 MIMO (proposed) antennas.

The channel capacity obtained from Fig. 13 and evaluated at SNR of 20 dB for the MIMO antenna to plot the CDF curves ^30^ in three different cases (Fig. 14). From the curve, it can be seen that the proposed antenna performs well since the CDF values of the proposed antenna are very close to the uncorrelated MIMO antenna (ideal case).

## Bending analysis of the 4-port MIMO antenna

**Figure 15:**
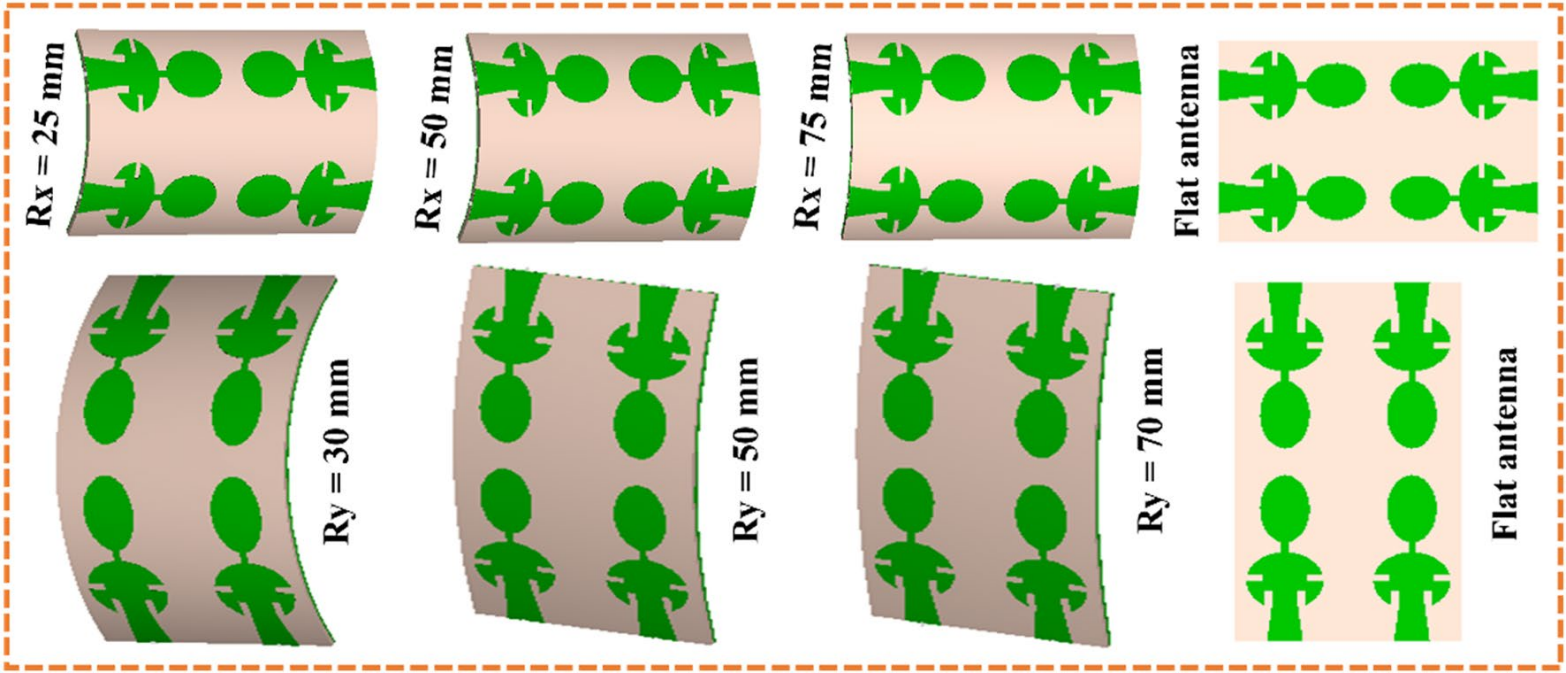
Bending analysis at different radii along x- and y-directions.

**Figure 16:**
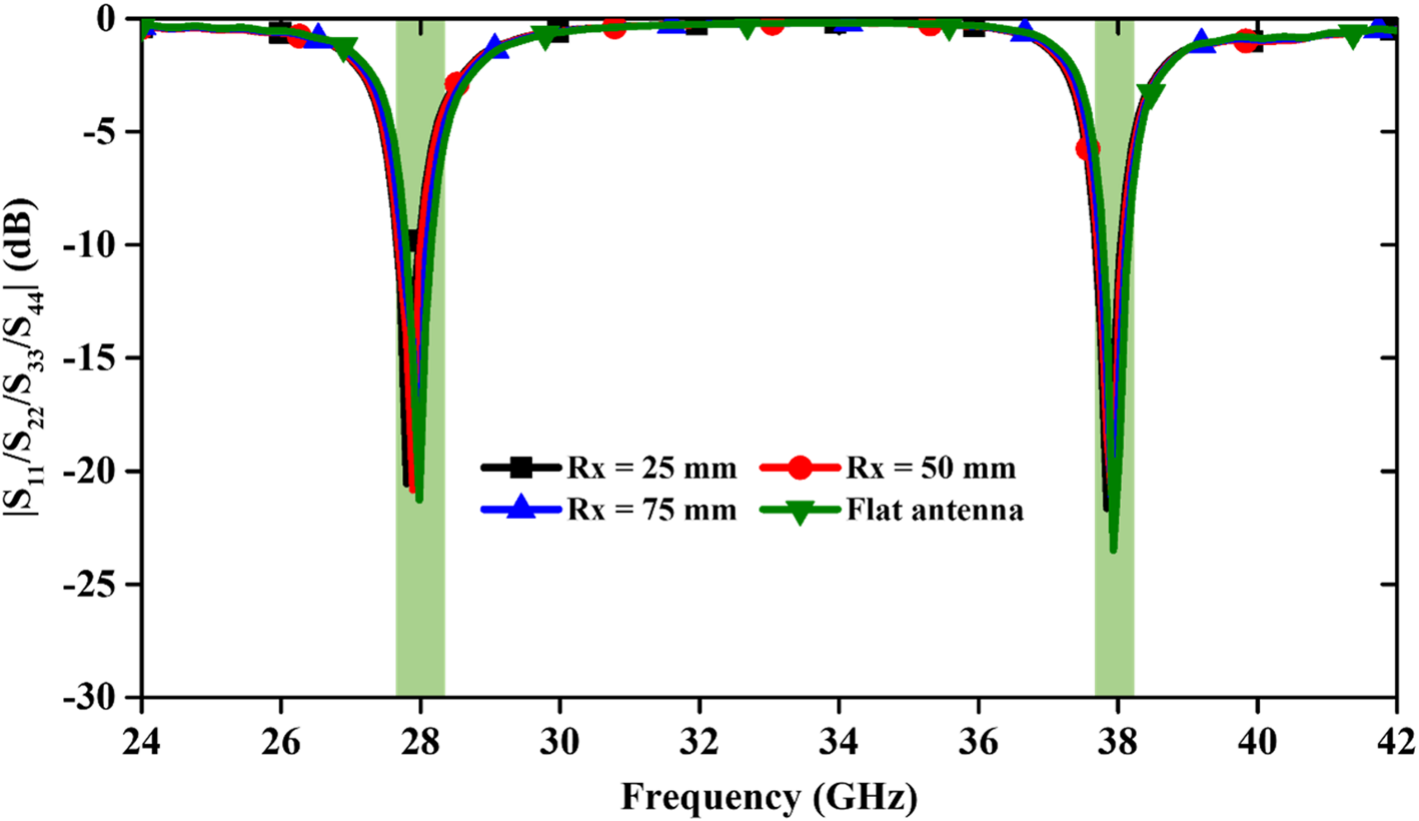
S-parameter variation with different bending radii along x-axis.

**Figure 17:**
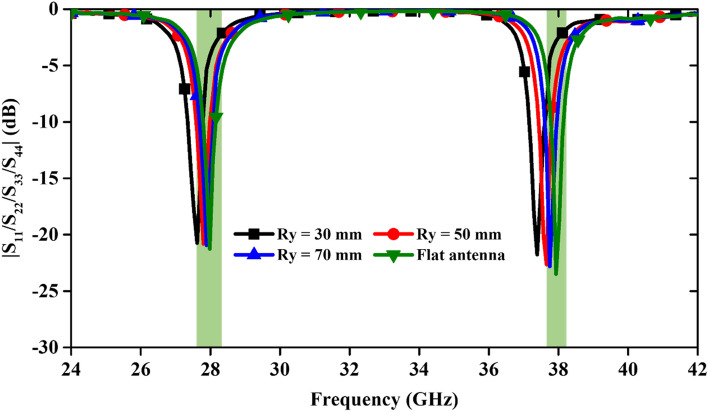
S-parameter variation with different bending radii along y-axis.

This section has analyzed the bending effect of the antenna to study its feasibility for on-body wearable applications. The MIMO antenna is bent along the x-direction with the bending radii (Rx) of 25, 50 and 75 mm, and along the y-direction it is bent with radii (Ry) of 30, 50 and 70 mm (Fig. 15). The S-parameter curves are demonstrated in Figs. 16 and 17 for the different bending radii of Rx and Ry, respectively. We have not shown the isolation curve because it was negligibly affected. From Fig. 16, it can be seen that the operating resonance frequencies are minutely affected due to the bending of the antenna along x-axis. When the antenna is deformed maximally along the y-axis (at Ry = 30 mm), resonant frequencies shift to the lower side.

## SAR (specific absorption rate) analysis

**Figure 18:**
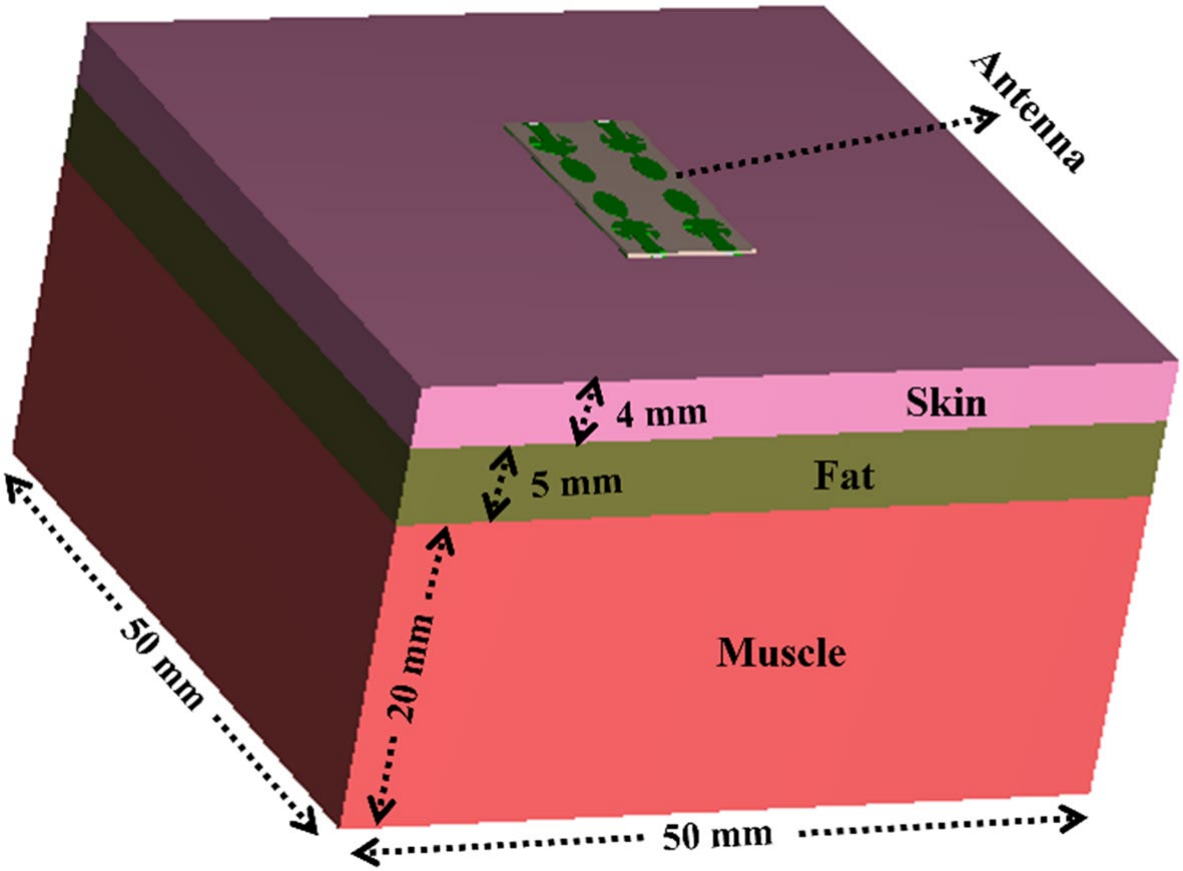
Multi-layer (skin, fat, and muscle) canonical body model.

SAR is defined as the EM (Electromagnetic) power absorbed by the tissues per unit mass. According to the International Commission on Non-Ionizing Radiation Protection (ICNIRP) and IEEE C95.1-2019 standard ^32^, for on-body application, the average SAR value should be below 1.6 W/kg and 2.0 W/kg (for 1 g and 10 g of tissues respectively). For the safety of the biological tissues, the SAR is calculated when the proposed mm-wave MIMO antenna is operating on the multi-layer (skin, fat, and muscle) canonical body model (shown in Fig. 18) at 28 GHz and 38 GHz.

**Figure 19:**
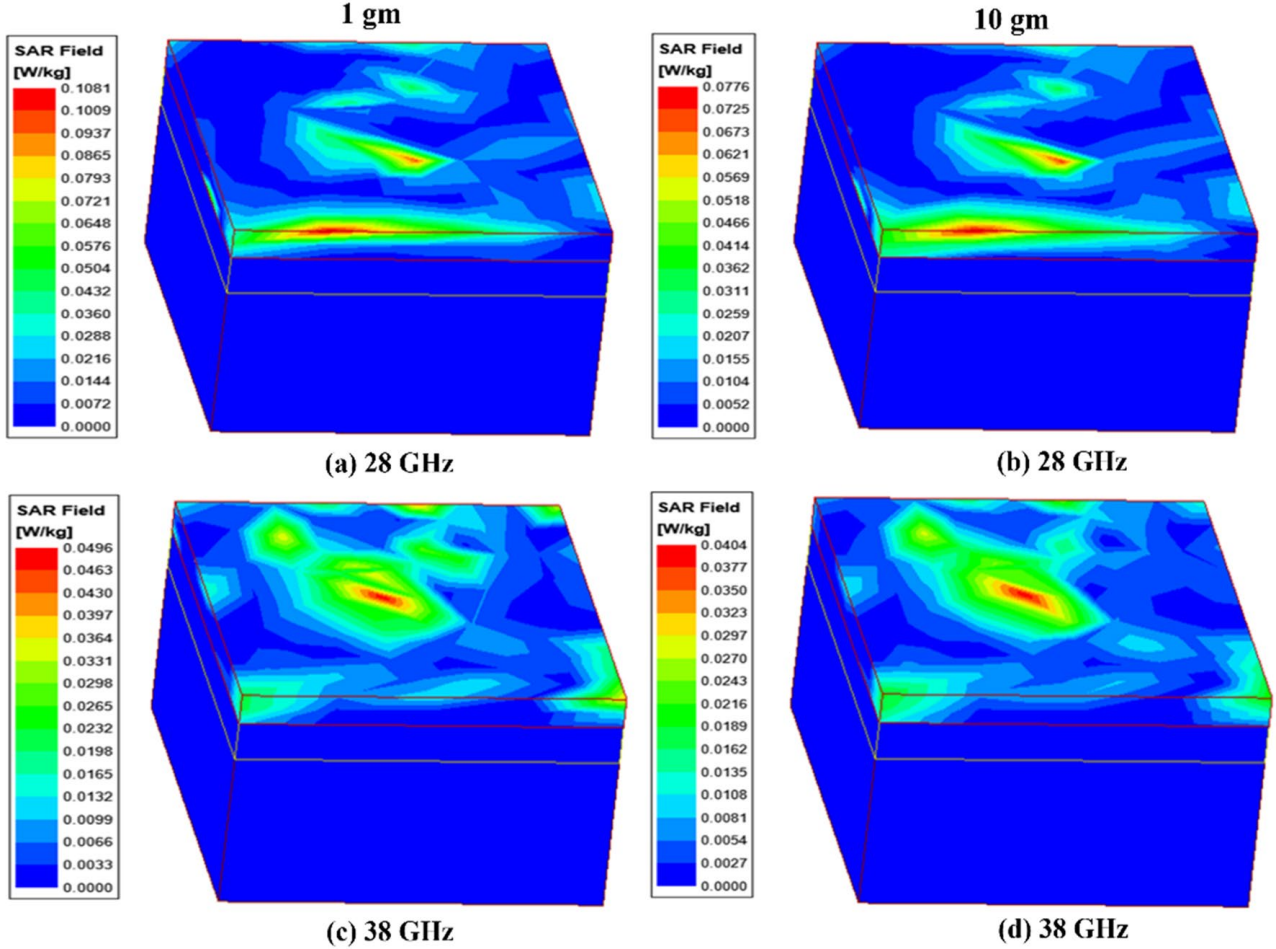
SAR of the mm-wave MIMO antenna on the multilayer body model.

To evaluate the SAR, the antenna is placed 5.0 mm above the multilayer canonical body model in the simulator. The electrical properties of the biological tissues are given in Table 3^33^. The phantom body model of 50 × 50 mm^2^ constitutes the muscles, fat and skin of thickness 20, 5 and 4 mm, respectively (Fig. 19). Average SAR (ASAR) for 1 gm and 10 gm tissues are presented in Table 4 (at 50 mW of the input power). The maximum average SAR at 28 GHz for 1 g/10 g values are 0.11/0.08 W/kg and 727.27/1250.00 mW whereas, at 38 GHz, the ASAR values are 0.05/0.04 W/kg and 1600/2500 mW, respectively.

**Table 3:** Electrical properties of body parts ^33^.

	28 GHz		38 GHz	
	Permittivity $\left({\varepsilon }_{r}\right)$	Conductivity $\left(\sigma \right)$	Permittivity $\left({\varepsilon }_{r}\right)$	Conductivity $\left(\sigma \right)$
Skin	16.6	25.8	12.3	31.0
Fat	6.09	5.04	5.33	6.36
Muscle	24.4	33.6	19.1	41.8

**Table 4:** Maximum average SAR of the proposed mm-wave MIMO antenna.

Frequency (GHz)	SAR (W/kg) (input power = 50 mW)		Maximum acceptable power (mW)		Maximum acceptable power (dBm)	
	1 g	10 g	1 g	10 g	1 g	10 g
28	0.11	0.08	727.27	1250.00	28.62	30.97
38	0.05	0.04	1600.00	2500.00	32.04	33.98

### Link budget analysis

The proposed MIMO antenna is designed for mm-wave wearable applications. Thus, it is important to study the Link Margin (LM) so that the distance for effective communication of the designed 4-port MIMO antenna can be evaluated ^34,35^. The link budget parameters are given in Table 5.

**Table 5:** Link budget parameters.

Transmitter		
	Operating frequency (GHz)	28/38
${G}_{t}$	Antenna gain (dBi)	4.15/7.73
${P}_{t}$	Transmitted power (dBm)	10
	EIRP (dBm)	14.15/17.73

Receiver		
${G}_{r}$	Receiver antenna gain (dBi)	4.1
${T}_{0}$	Ambient temperature (K)	293
$k$	Boltzmann constant	1.38 × 10^–23^
${N}_{0}$	Noise power density (dB/Hz)	− 203.9

Signal quality		
${B}_{r}$	Bit rate (Mb/s)	1, 10, 50, 100
	Bit error rate	1 × 10^–5^
${E}_{b}/{N}_{0}$	Ideal PSK (dB)	9.6
${G}_{c}$	Coding gain (dB)	0
${G}_{d}$	Fixing deterioration (dB)	2.5

**Figure 20:**
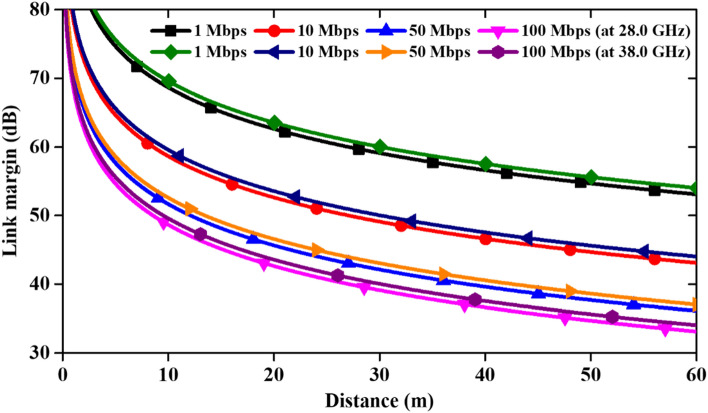
Link margin between transmitting and receiving antenna at 28/38 GHz.

Figure 20 shows that the LM decreases as communication distance increases. From the curve it can be observed that the link margin is minimum when the data rate is maximum. The proposed antenna can transmit/receive at the data rate of 100 Mbps up to a communication distance of 60 m with LM of more than 33 dB (for both the resonant frequencies of 28/38 GHz).

## Conclusion

In this paper, a coupled resonator approach is applied to design a flexible 4-port MIMO antenna operating in mm-wave frequency range. The MIMO element consists of circular and elliptical-shaped patches connected by narrow metallic strips that resonate at 28 and 38 GHz. The operating frequencies of the proposed MIMO antenna can be tuned by the slots etched in the radiating patch and in the ground plane. We have evaluated the diversity parameters to check the usefulness of the proposed antenna for 5G applications. The 1 g/10 g SAR values are also calculated on the multilayer phantom model and found below the predefined safe values. The designed 4-port MIMO antenna can communicate up to 60 m of distance at the high data rate of 100 Mbps at both the operating frequencies of 28/38 GHz. Therefore, this antenna can be very effective for high data rate mm-wave 5G communication and wearable electronics.

## Data Availability

Data is provided within the manuscript.
